# Anti-PD-1 antibody significantly increases therapeutic efficacy of *Listeria monocytogenes (Lm)-*LLO immunotherapy

**DOI:** 10.1186/2051-1426-1-15

**Published:** 2013-08-29

**Authors:** Mikayel Mkrtichyan, Namju Chong, Rasha Abu Eid, Anu Wallecha, Reshma Singh, John Rothman, Samir N Khleif

**Affiliations:** 1 Cancer Center, Georgia Regents University, 1120 15th Street, Augusta GA 30192, USA; 2 National Cancer Institute, NIH, Vaccine Branch, Bethesda, MD 20892, USA; 3 Advaxis Inc., Princeton, NJ 08540, USA

**Keywords:** PD-1, Immunotherapy, Listeria-based vaccine, Combinational immunotherapy

## Abstract

**Background:**

One of the significant tumor immune escape mechanisms and substantial barrier for successful immunotherapy is tumor-mediated inhibition of immune response through cell-to-cell or receptor/ligand interactions. Programmed death receptor-1 (PD-1) interaction with its ligands, PD-L1 and PD-L2, is one of the important strategies that many tumors employ to escape immune surveillance. Upon PD-Ls binding to PD-1, T cell receptor (TCR) signaling is dampened, causing inhibition of proliferation, decreased cytokine production, anergy and/or apoptosis. Thus PD-Ls expression by tumor cells serves as a protective mechanism, leading to suppression of tumor-infiltrating lymphocytes in the tumor microenvironment. *Lm*-LLO immunotherapies have been shown to be therapeutically effective due to their ability to induce potent antigen-specific immune responses. However, it has been demonstrated that infection with *Lm* leads to up-regulation of PD-L1 on mouse immune cells that can inhibit effector T cells through PD-1/PD-L1 pathway.

**Methods:**

Therapeutic and immune efficacy of Listeria-based vaccine (Lm-LLO-E7) in combination with anti-PD-1 antibody was tested in E7 antigen expressing TC-1 mouse tumor model. Tumor growth, survival, as well as peripheral and tumor-infiltrating immune cell profiles after immunotherapy were assessed.

**Results:**

Here we demonstrate that the combination of an *Lm*-LLO immunotherapy with anti-PD-1 antibody that blocks PD-1/PD-L1 interaction, significantly improves immune and therapeutic efficacy of treatment in TC-1 mouse tumor model. Importantly, we show that in addition to significant reduction of regulatory T cells (Treg) and myeloid-derived suppressor cells (MDSC) in both spleen and tumor microenvironment that are mediated solely by the *Lm*-LLO immunotherapy, the addition of anti-PD-1 antibody to the treatment results in significant increase of antigen-specific immune responses in periphery and CD8 T cell infiltration into the tumor. As a result, this combinational treatment leads to significant inhibition of tumor growth and prolonged survival/complete regression of tumors in treated animals.

We also demonstrate that *in vitro* infection with Lm results in significant upregulation of surface PD-L1 expression on human monocyte-derived dendritic cells suggesting the translational capacity of this finding.

**Conclusions:**

Our findings demonstrate that combination of *Lm*-LLO-based vaccine with blocking of PD-1/PD-L1 interaction is a feasible approach with clinical translation potential that can lead to overall enhancement of the efficacy of anti-tumor immunotherapy.

## Background

*Listeria monocytogenes* (*Lm*) is a Gram-positive facultative anaerobic intracellular bacterium that has been extensively studied as a vaccine vector for several diseases. The unique intracellular life cycle of *Lm* in an antigen-presenting cell (APC) allows antigen to be processed and presented in the context of both MHC I and II molecules, resulting in strong CD8^+^ and CD4^+^ T cell-mediated immune responses [1]. The listeriolysin-O (LLO) protein is the major virulence factor of *Lm* responsible for the lysis of the phagolysosome vacuole. Recently, LLO has been shown to be PAMP-like molecule by stimulating production of proinflammatory cytokines and inducing maturation of antigen-presenting cells [2]. Previously published reports have shown that genetically fusing an HPV16-E7 to a non-functional truncated form of LLO enhances the immunogenicity of antigens, as compared to the antigen expressed alone in the same system [3]. Furthermore, this enhanced immunogenicity correlates with a better therapeutic efficacy against established tumors [3,4].

One of several mechanisms of tumor-mediated immune suppression is the expression of co-inhibitory molecules by tumor. Upon engagement to their ligands these molecules can suppress effector lymphocytes in the periphery and in the tumor microenvironment [5,6]. The PD-1 is one of the central signaling molecules that may inhibit T cell immunity when bound to its ligands (PD-L1 or PD-L2) by inducing T cell apoptosis and anergy [7]. PD-1 is expressed on the surface of activated lymphocytes and myeloid cells [8]. PD-L1 is expressed on activated T cells, B cells, dendritic cells and macrophages, in addition to a wide range of non-hematopoietic cells [9]. PD-L1 is upregulated on numerous human tumors, and its expression has been shown to inversely correlate with survival in different types of cancer [10-15]. The expression of PD-L2 on various tumor cells was also demonstrated [16,17].

It has been shown that tumor eradication can be enhanced by PD-L1/PD-1 blockade [18-23]. Recently we demonstrated that the combination of PD-1 targeting with vaccine and low-dose cyclophosphamide significantly enhances antigen-specific immune responses, decreases tumor burden and increases survival of treated mice [22,24].

Interestingly, in addition to significant immune and therapeutic potency of listeria-based immunotherapy [3,4], it has been demonstrated that infection with listeria lead to up-regulation of PD-L1 on immune cells [25].

Thus, we hypothesized that combination of Listeria-based vaccine with blockade of PD-1/PD-L interaction could improve the overall anti-tumor efficacy of immunotherapy.

Here we tested the therapeutic efficacy and immune mechanisms of anti-PD-1 antibody combined with listeria expressing LLO and E7 antigen (Lm-LLO-E7) in TC-1 tumor model.

## Methods

### Animals, cells lines, vaccine and other reagents

Six to eight weeks old female C57BL6 mice were purchased from NCI Frederick and kept under pathogen-free conditions. Mice were cared for under protocols approved by the NCI Animal Care and Use Committee. TC-1 cells that were derived by co-transfection of human papillomavirus strain 16 (HPV16) early proteins 6 and 7 (E6 and E7) and activated ras oncogene to primary C57BL/6 mouse lung epithelial cells were obtained from ATCC (Manassas, VA), and cells were grown in RPMI 1640 supplemented with 10% FBS, penicillin and streptomycin (100 U/ml each) and L-glutamine (2 mM) at 37°C with 5% CO_2_. Listeria vaccine vectors with or without human papilloma virus-16 (HPV-16) E7 (Lm-LLO and Lm-LLO-E7) provided by Advaxis Inc. were generated as described previously [3]. Both Lm-LLO and Lm-LLO-E7 were injected intraperitonealy (i.p.) at 5 × 10^6^ CFU/mouse dose. The anti-PD-1 monoclonal antibody was obtained from CureTech (Israel) and was injected intravenously (i.v.) at a dose of 50 μg/mouse. All fluorescently labeled antibodies and appropriate isotype controls used for flow cytometry were purchased from BD Biosciences (San Jose, CA) or eBiosciences (San Diego, CA).

### Mouse and human dendritic cell isolation, purification and analysis of PD-L1 expression

Mouse dendritic cells (DC) were isolated and purified from bone marrow as we described earlier [26]. To obtain human DC, monocytes were isolated from healthy adult blood donors (National Institute of Health, Blood bank). Briefly, peripheral blood mononuclear cells (PBMC) were isolated from gradient centrifugation using Ficoll-Paque Plus (Amersham Biosciences) and, after washing, allowed to adhere to tissue culture plates for 2 h at 37°C. Non-adherent cells were removed by washing, and the adherent monocytes were cultured in a plate at 37°C, 5% CO2 in complete RPMI 1640 consisting of RPMI 1640, 2 mM L-glutamine, penicillin (100 U/ml), streptomycin (100 ug/ml), 10 mM HEPES, 10% fetal bovine serum, 10 mM nonessential amino acids, 1 mM sodium pyruvate, and 5 × 10^-5^ M 2-mercaptoethanol. Cells were cultured in the presence of GM-CSF (1000 U/ml) and IL-4 (500 U/ml) for 4 days to become immature DCs. GM-CSF and IL-4 were added again along with fresh medium on day 3. The DC viability in cultures was assessed using the trypan blue exclusion protocol. Trypan blue-negative cells were considered alive. After culturing DCs from monocytes for 4–5 days, DCs were collected and transferred to 6 well plate (1 × 10^6^ cells/ml). Different concentrations of Lm-LLO or Lm-LLO-E7 were added to DCs culture (0, 10^7^, 10^8^, and 10^9^ CFU/ml) for an hour followed by adding gentamicin (50 ug/ml) to kill listeria, and cultured for 48 hr.

Both mouse and human DCs were stained with appropriate fluorescently labeled anti-PD-L1 antibody (PE anti-mouse PD-L1 and FITC anti-human PD-L1). Isotype-matched mAbs were used as negative controls. The stained cells were analyzed using FACSCalibur cytometer and CellQuest software (BD Biosciences).

### Tumor implantation and treatment

The therapeutic experiments aimed to analyze tumor growth and survival were performed as described earlier [22,24]. Briefly, mice were implanted with 50,000 TC-1 cells/mouse subcutaneous (s.c.) in the right flank on day 0. On day 8 (tumor size ~3–4 mm in diameter), animals from appropriate groups (5 mice per group) were injected i.p. with Lm-LLO or Lm-LLO-E7 with or without anti-PD-1 Ab i.v. Mice were treated with vaccine and anti-PD-1 Ab one more time on day 15 after tumor implantation. Another group of mice remained non-treated. Tumors were measured every 3–4 days using digital calipers, and tumor volume was calculated using the formula V = (W^2^ × L) /2, whereby V is volume, L is length (longer diameter) and W is width (shorter diameter). In these experiments mice were sacrificed when mice became moribund, tumors were ulcerated or tumor volume reached 1.5 cm^3^.

In immunologic experiments same groups of mice were treated similarly, except mice were sacrificed six days after the second treatment, on day 21. Spleens and tumors were isolated and analyzed for antigen-specific immune responses, CD8 T cells, Tregs and myeloid derived suppressor cells (MDSC).

### Analysis of antigen-specific cellular immune responses, Tregs, MDSC in periphery and tumors

ELISPOT was used to detect IFNγ production in E7-restimulated (10 μg/ml) splenocyte cultures from treated and control mice, as suggested by the manufacturer (BD Biosciences, San Jose, CA). A CTL Immunospot Analyzer (Cellular Technology Ltd., Shaker Heights, OH) was used to analyze spots. The number of spots from irrelevant peptide (hgp 100_25–33_- KVPRNQDWL-Celtek Bioscience, Nashville, TN) re-stimulated splenocytes were subtracted from E7-restimulated cultures.

Tumor samples were processed using GentleMACS Dissociator and the solid tumor homogenization protocol, as suggested by the manufacturer (Miltenyi Biotec, Auburn, CA). The number of tumor-infiltrating CD8^+^, CD4^+^Foxp3^+^ (Treg) and CD11b^+^Gr-1^+^(MDSC) cells were analyzed within CD45^+^ hematopoietic cell population using flow cytometry assay as we described earlier [22,26]. The level of Treg cells and MDSC was also evaluated in spleens of tumor-bearing treated and control mice using the same flow cytometry assay.

### Statistical analysis

All statistical parameters (average values, SD for PD-L1 expression on DC, tumor volumes, ELISPOT and peripheral and tumor-infiltrating cell analysis) and statistical significance between groups (for peripheral and tumor-infiltrating cell analysis) were calculated using GraphPad Prism Software (San Diego, CA). Statistical significance between groups was determined by one-way ANOVA with Tukey’s multiple comparison post-test (*P <* 0.05 was considered statistically significant).

## Results

### Infection of murine DC with Lm-LLO and Lm-LLO-E7 upregulates surface PD-L1 expression

**Figure 1:**
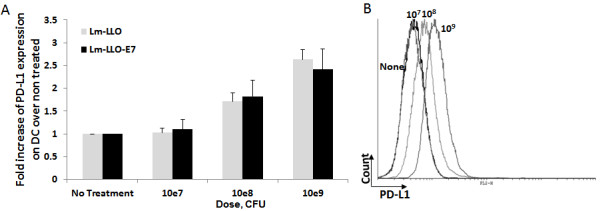
**Lm-LLO and Lm-LLO-E7 infection upregulates PD-L1 expression on mouse DC surface. A**. Fold increase of PD-L1 expression on bone marrow derived mouse DC after treatment with different concentrations of Lm-LLO or Lm-LLO-E7 over non-treated control. **B**. Representative histogram from one out of three independent experiments.

It was previously demonstrated that mouse splenocytes infection with Lm results in significant upregulation of PD-L1 expression on the majority of cells, and that the level of PD-L1 expression was highest among CD11c^+^ DC [25]. Considering the importance of DC in priming immune response and inhibitory role of PD-1/PD-L1 interaction, we decided first to analyze the effect of Lm-LLO and Lm-LLO-E7 on the PD-L1 expression on DC. To avoid the influence of cell-cell interactions within the mixed cell population on the accuracy of results, we tested the effect of different concentrations of Lm-LLO and Lm-LLO-E7 on PD-L1 expression on the surface of purified CD11c^+^ DCs. As shown in Figure 1, both Lm-LLO and Lm-LLO-E7 significantly upregulate PD-L1 expression at 10^8^ and 10^9^ CFU/ml doses in a dose dependent manner. Importantly, there were no differences detected between Lm-LLO- and Lm-LLO-E7-induced PD-L1 upregulation on DC at any of the tested doses (Figure 1), indicating that this effect is antigen-independent.

### Anti-PD-1 enhances therapeutic efficacy of Lm-LLO-E7 vaccine

**Figure 2:**
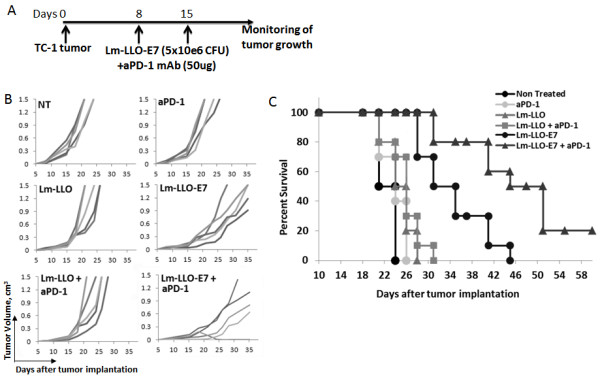
**Addition of anti-PD-1 Ab to Lm-LLO-E7 enhances therapeutic potency of treatment. A**. Treatment schedule. **B**. Tumor volumes of individual mice for each treatment measured every 3–4 days. **C**. The Kaplan-Meier plot depicts overall survival. Similar results were obtained from three independent experiments.

After confirming the effect of Lm-LLO and Lm-LLO-E7 on upregulation of PD-L1 expression on DC, and considering the inhibitory effect of PD-1/PD-L1 interaction we hypothesize that combination of PD-1/PD-L1 blockade with Listeria-based vaccine could improve the anti-tumor efficacy of immunotherapy. To test this hypothesis we evaluated the effect of anti-PD-1 Ab and Lm-LLO-E7 combination on tumor growth and survival of mice in TC-1 tumor model based on E7-expressing lung epithelial cells. We deliberately used a low dose of Lm-LLO-E7, delayed treatment schedule and implanted a high number of tumor cells in order to minimize the effect of vaccine alone. Mice were implanted with 50,000 TC-1 cells s.c. on day 0, and on days 8 and 15 after tumor implantation mice were injected with Lm-LLO-E7 or Lm-LLO with or without anti-PD-1 Ab (Figure 2A). Another group of mice remained non-treated.

While Lm-LLO-E7 vaccine alone resulted in slight inhibition of tumor growth, Lm-LLO-E7/anti-PD-1 combination significantly slowed tumor growth (Figure 2B) and resulted in prolonged survival and complete tumor regression in 20% of treated mice (Figure 2C).

These experiments reveal that combination of anti-PD-1 Ab with Lm-LLO-E7 vaccine is a feasible strategy resulting in tumor growth inhibition and improved survival even at stringent conditions that were used in these experiments.

### Combination of anti-PD-1 Ab and Lm-LLO-E7 significantly enhances antigen-specific immune responses and CD8 T cell infiltration into the tumor

**Figure 3:**
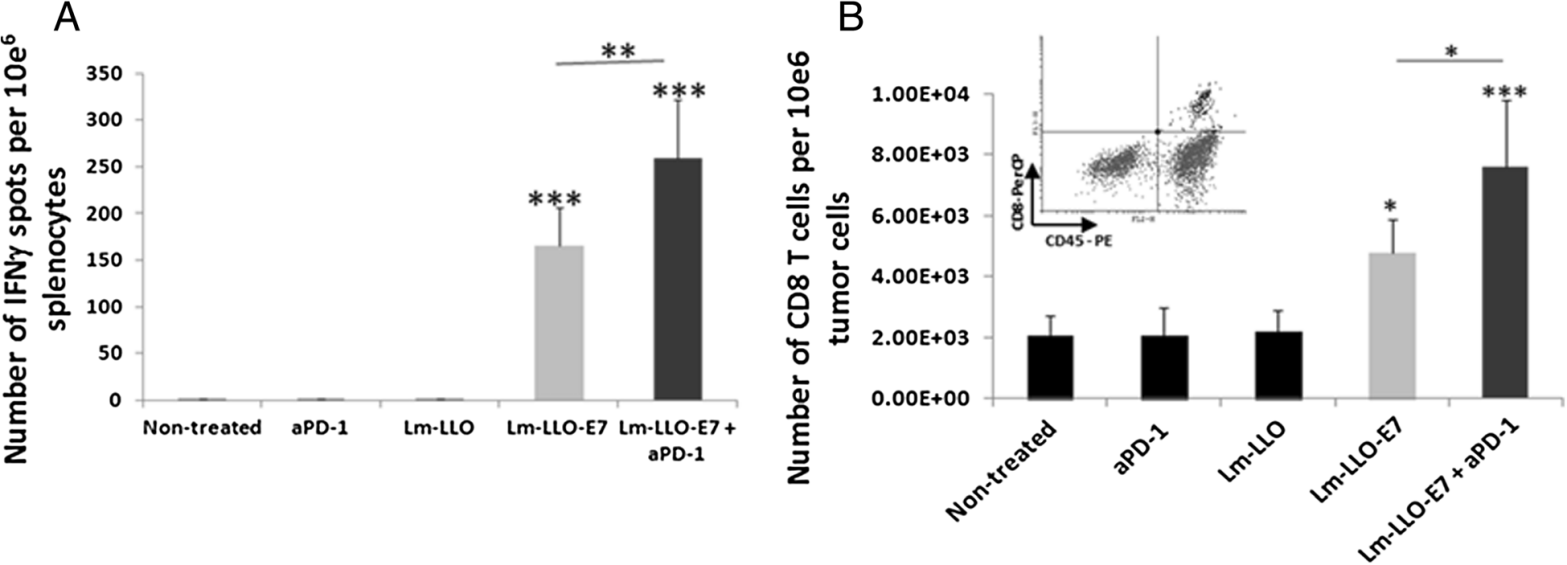
**Addition of anti-PD-1 Ab to Lm-LLO-E7 enhances antigen-specific immune responses and increases the level of tumor-infiltrated CD8 T cell.** C57BL/6 mice (n = 5 per group) were treated as on Figure 2A, except on day 21 after tumor implantation mice were sacrificed. **A**. IFNγ production in the presence or absence of E7 peptide was analyzed in single-cell suspension obtained from spleens. Values represent number of spots from E7-re-stimulated culture minus that from irrelevant antigen re-stimulated culture ± SD. **B**. The absolute numbers of infiltrated CD45^+^CD8^+^ T cells were standardized per 10e6 of total tumor cells and presented as mean values ± SD. *P < 0.05, **P < 0.01 and ***P < 0.001. Similar results were obtained from two independent experiments.

To define the immune mechanism and evaluate the immunologic efficacy of Lm-LLO-E7/anti-PD-1 Ab combination we next assessed the levels of antigen-specific IFNγ-producing cells in spleens from treated tumor-bearing mice and tumor-infiltrated CD8 T cells. Mice were implanted with TC-1 cells and treated as described above for therapeutic experiments, except, six days after the second treatment mice were sacrificed and spleens and tumors were harvested. Analysis of E7-specific IFNγ-producing cells was performed using a standard ELISPOT assay. As expected, treatment with Lm-LLO-E7 alone induced significant levels of IFNγ-producing E7-specific cells compared to controls (P < 0.001). Notably, addition of PD-1/PD-L1 blockade with anti-PD-1 Ab, to Lm-LLO-E7 resulted in further significant increase in antigen specific immune response when compared to Lm-LLO-E7 alone (P < 0.01) (Figure 3A).

To further determine the mechanism by which combining Lm-LLO-E7/anti-PD-1 Ab exerts its therapeutic effect, we tested the influence of treatment on tumor-infiltrated CD8 T cells. Tumor-infiltrated CD8 T cells were tested on day 21 post tumor implantation in mice treated as described above. As expected, Lm-LLO-E7 and Lm-LLO-E7/anti-PD-1 Ab showed a significant increase in tumor-infiltrated CD8 T cells compared to control groups (P < 0.05 for Lm-LLO-E7 alone and P < 0.001 for Lm-LLO-E7/anti-PD-1 Ab) (Figure 3B). Similar to peripheral immune response, addition of anti-PD-1 Ab to Lm-LLO-E7 treatment resulted in significant increase in CD8 T cell tumor infiltration compared to Lm-LLO-E7 alone (P < 0.05) (Figure 3B).

### Lm-LLO treatment significantly reduces both splenic and tumor-infiltrated MDSC and Treg cells regardless of presence of antigen or anti-PD-1 Ab

**Figure 4:**
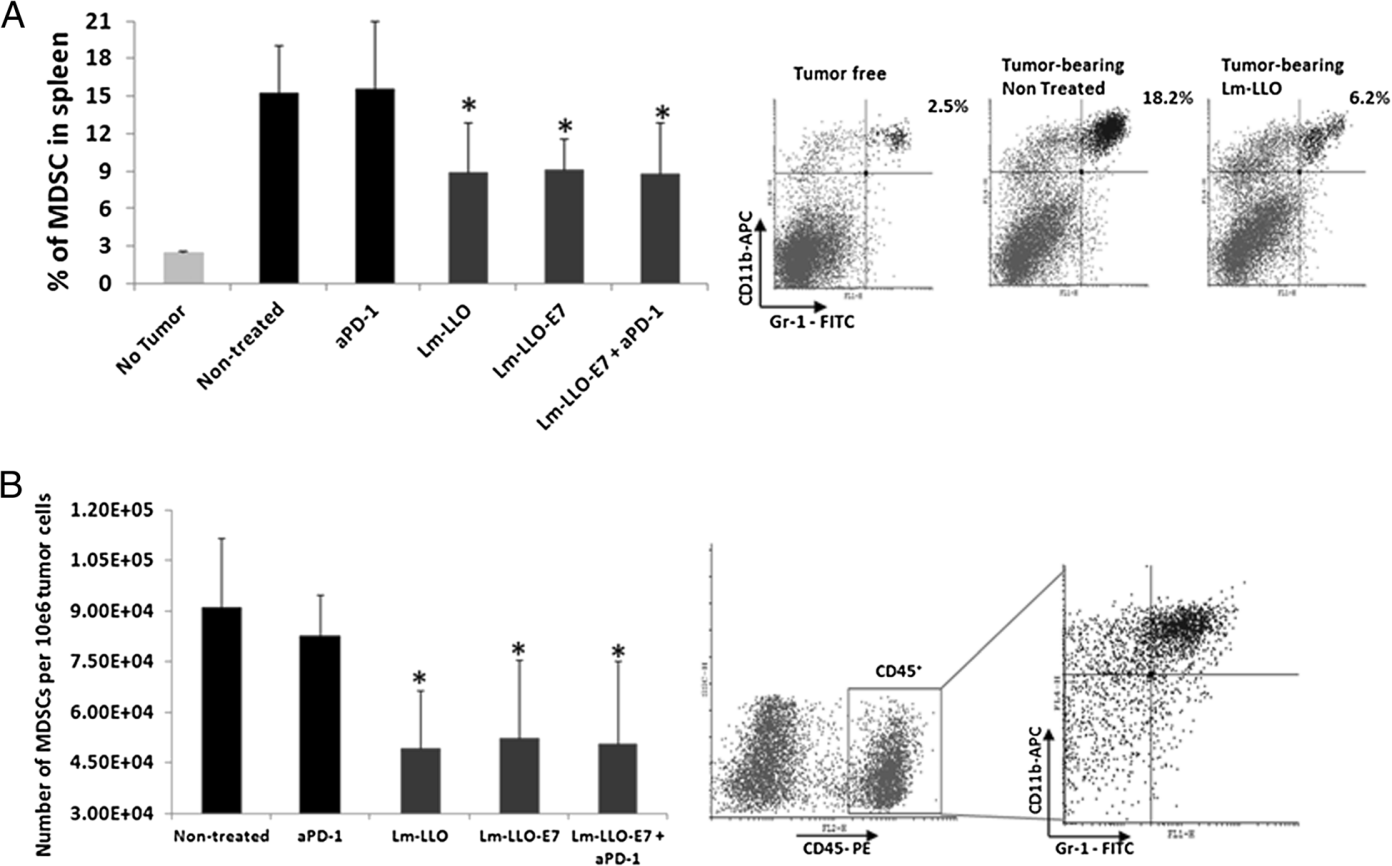
**Lm-LLO treatment decreases the levels of splenic and tumor infiltrating MDSC. A**. The percentage of splenic CD11b^+^Gr-1^+^ MDSC from treated and control mice C57BL/6 mice (n = 5). **B**. The absolute numbers of infiltrated CD45^+^CD11b^+^Gr-1^+^ MDSC standardized per 10e6 of total tumor cells are presented as mean values ± SD. *P < 0.05. Similar results were obtained from two independent experiments.

**Figure 5:**
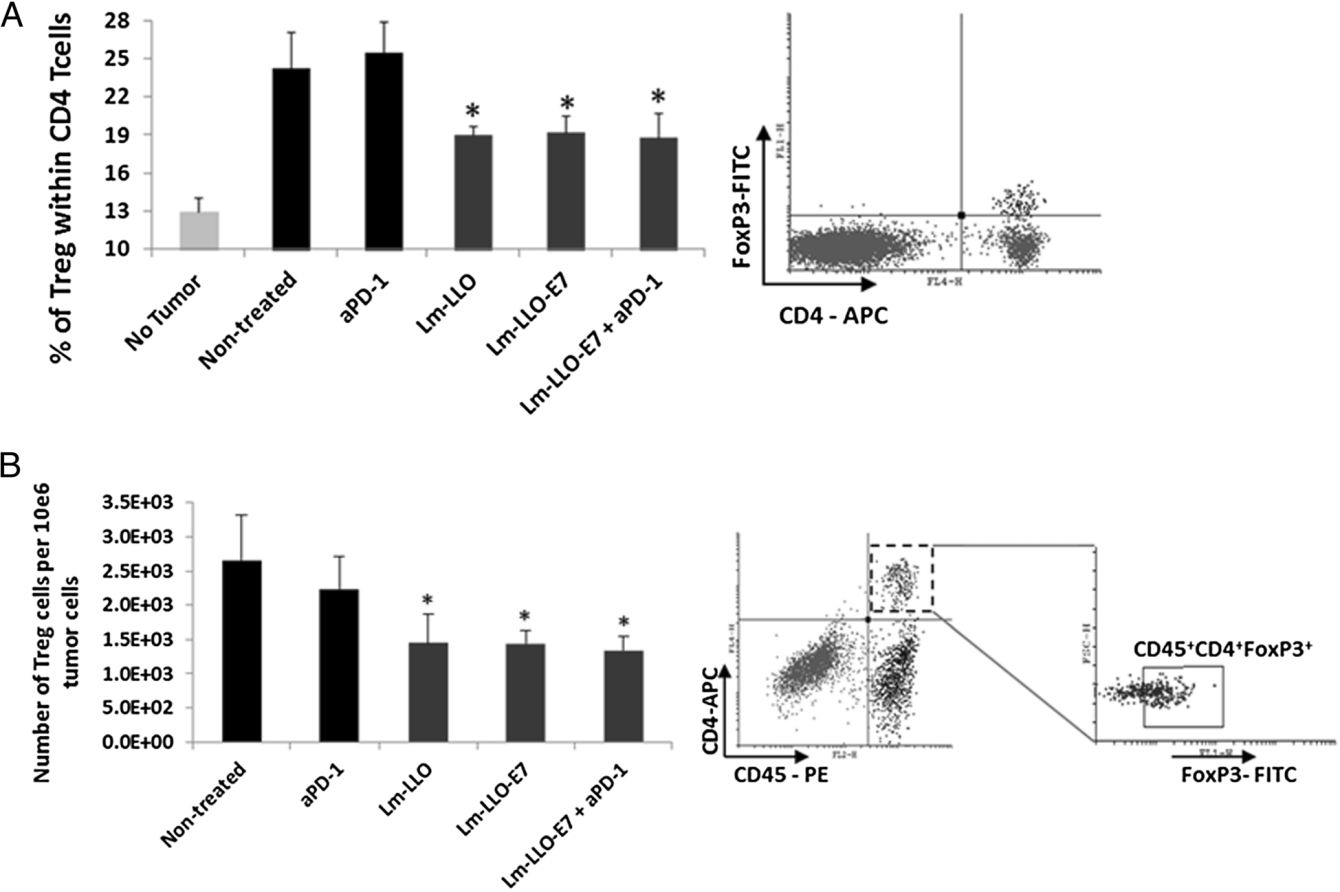
**Lm-LLO treatment decreases the levels of splenic and tumor infiltrating Treg cells. A**. The percentage of CD4^+^FoxP3^+^ Treg cells within CD4^+^ cell population of splenocytes from experimental and control groups. **B**. The absolute numbers of infiltrated CD45^+^CD4^+^FoxP3^+^ Treg cells standardized per 10e6 of total tumor cells are presented as mean values ± SD. *P < 0.05. Similar results were obtained from two independent experiments.

Two cell subsets with profound immune response inhibitory activity are MDSC and Treg cells. Accordingly, we analyzed these subsets both in periphery and within tumor microenvironment to understand the impact of Lm-LLO-E7/anti-PD-1 Ab combinational treatment. Spleens and tumors harvested six days after second vaccination were assessed for percent (spleen) and actual numbers (tumors) of MDSC and Treg cells. While the percent of MDSC in spleens of tumor-free animals is about 2.5%, in presence of tumor this percent significantly increases (~15%) (Figure 4A). Interestingly, treatment with Lm-LLO, regardless of presence of E7 antigen or anti-PD-1 treatment, significantly decreases the levels of MDSC in spleens compared to control animals (P < 0.05) (Figure 4A). Similarly, numbers of tumor-infiltrated MDSC also were significantly decreased after treatment with Lm-LLO, Lm-LLO-E7 and Lm-LLO-E7/anti-PD-1 Ab treatment (Figure 4B). Importantly, Treg cells in both spleens (Figure 5A) and tumors (Figure 5B) were also slightly but significantly decreased in groups treated with Lm-LLO either alone or with E7 or anti-PD-1 Ab.

These data suggest that Lm-LLO is solely responsible for decrease of MDSC and Tregs in both spleens and tumors of treated mice, and that the addition of antigen or anti-PD-1 antibody does not affect levels of these cells.

### Infection of human DC with Lm-LLO also leads to upreguation of surface PD-L1 expression

**Figure 6:**
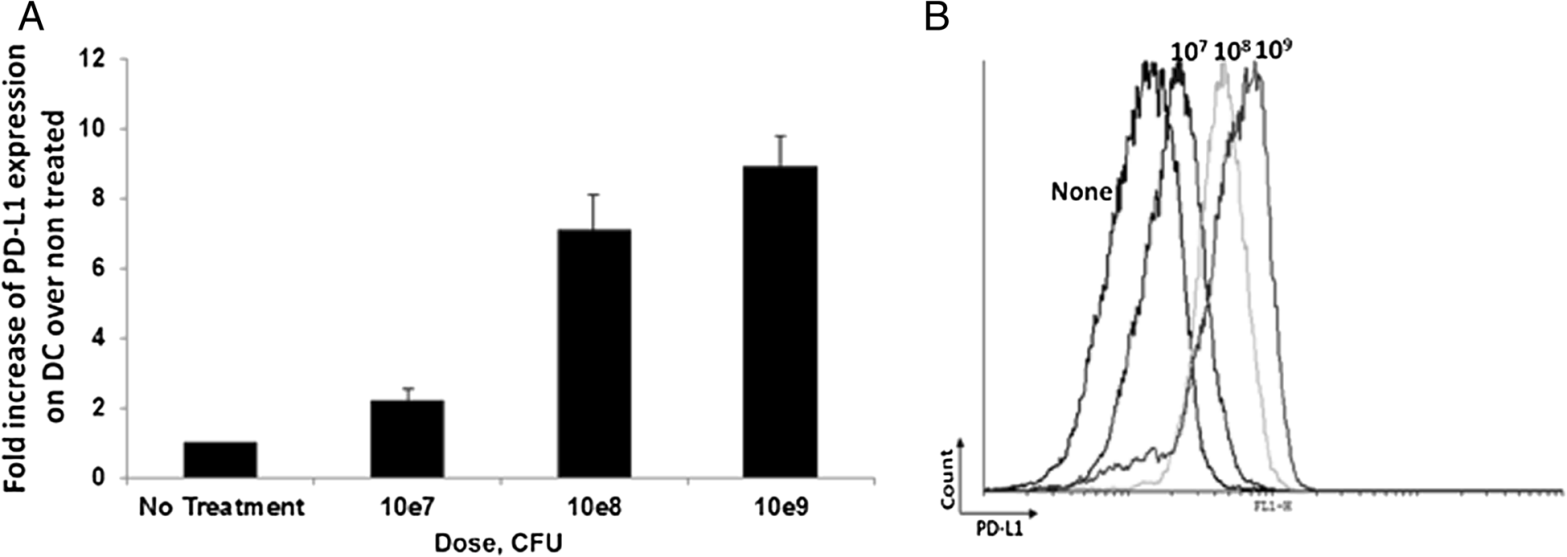
**Lm-LLO infection upregulates PD-L1 expression on monocyte-derived human DC surface. A**. Fold increase of PD-L1 expression on human DC after treatment with different concentrations of Lm-LLO over non-treated control. **B**. Representative histogram of PD-L1 expression on human DC treated with different concentrations of Lm-LLO. Similar results were obtained from three independent experiments.

After demonstrating the therapeutic efficacy and immune mechanism by which Lm-LLO-E7/anti-PD-1 Ab combination exerts anti-tumor effect, we decided to test if Lm-LLO also affect the levels of PD-L1 expression on human DC and so as, to understand if our findings could be translated into the clinic. Monocyte-derived human DC were isolated from PBMC of healthy volunteers as described in Methods section. Human DC were infected with different concentrations of Lm-LLO and Lm-LLO-E7. We found that, similar to murine DC, both Lm-LLO and Lm-LLO-E7 infection leads to significant upregulation of surface PD-L1 (Figure 6A and B and data not shown). As for murine DC, the PD-L1 upregulation on human DC was dose dependent. This finding suggests that combination of listeria-based vaccine with anti-PD-1 Ab could be a potent and clinically translatable immunotherapeutic approach.

## Discussion

The PD-1/PD-L1 engagement has been shown to dampen TCR signaling, decrease cytokine production by the T cell, thus, reducing proliferation, anergy and/or apoptosis [27,28]. Accordingly, PD-L1 expression by tumor cells serves a protective function that can lead to suppression of tumor-infiltrating effector lymphocytes thus, allowing tumor to escape immune surveillance [29-31]. On the other hand, in disease-free conditions, PD-1/PD-L1 interaction serves as an important factor in preventing autoimmunity [9,32].

It was previously demonstrated that Listeria-based tumor antigen delivery is a therapeutically potent strategy capable of inducing robust antigen-specific immune responses, inhibiting tumor growth and prolonging survival in pre-clinical mouse tumor models [3,4] and is currently in clinical trials. A Phase 1 study has been completed with Lm-LLO-E7 [33] and 4 Phase 2 clinical trials are active or about to be initiated [34].

Interestingly, despite the promising therapeutic potency of Listeria-based immunotherapies, it was shown that infection with listeria leads to up-regulation of PD-L1 on immune cells [25]. This observation allowed us to hypothesize, that combination of PD-1/PD-L1 blockade with Listeria-based immunotherapy will result in even more potent anti-tumor efficacy of treatment. To test this hypothesis we first confirmed the effect of Listeria infection on PD-L1 expression on both mouse and human DCs and then evaluated the immune and therapeutic efficacy of Lm-LLO-E7 combination with anti-PD-1 Ab in mouse TC-1 tumor model. To minimize the effect of Listeria vaccine alone and thus, understand the impact of PD-1/PD-L1 interaction blockade, we used delayed treatment schedule and low dose of Lm-LLO-E7. We demonstrate that even at these stringent conditions addition of anti-PD-1 Ab to Listeria-based treatment results in improvement of tumor-growth inhibition and prolongation of survival and significantly stronger antigen-specific immune response compared to Listeria vaccine alone.

We showed previously that combination of anti-PD-1 Ab with peptide vaccine requires incorporation of single low dose of cyclophosphamide (CPM) treatment to decrease Treg cell numbers, and that potent therapeutic efficacy is seen only when all three components are combined [22,24]. Interestingly, here we found that while addition of anti-PD-1 Ab to Lm-LLO-E7 also significantly increases infiltration of CD8 T cells into the tumor, it does not affect the levels of neither peripheral nor tumor-infiltrated suppressor cells. Instead, surprisingly, Lm-LLO regardless of the presence of antigen or PD-1/PD-L1 interaction blockade, results in significant decrease of Treg cells and MDSC in both spleens and tumors of treated animals. While we previously demonstrated that Lm-LLO is able to decrease tumor-infiltrated Treg cells [34], here we demonstrate for the first time, to our knowledge, that Lm-LLO treatment results in decrease of MDSC. Although the mechanisms for both Lm-LLO-induced Treg cells and MDSC decrease are still unknown and currently under investigation in our laboratories, here we demonstrate that these events along with PD-1/PD-L1 interaction blockade with anti-PD-1 Ab lead to increased antigen-specific immune responses and thus, result in enhancement of overall anti-tumor efficacy of treatment.

## Conclusions

Overall, our findings demonstrate that combination of *Lm*-LLO-based vaccine with anti-PD-1 Ab leads to increased antigen-specific immune responses and tumor-infiltrating CD8 T cell, decrease in suppressor cells (Treg cells and MDSC) and as a result, leads to significant inhibition of tumor growth and prolonged survival/complete regression of tumors in treated animals. Thus, we show that combination of *Lm*-LLO-based vaccine with blocking of PD-1/PD-L1 interaction is a feasible and translatable approach that can lead to overall enhancement of the efficacy of anti-tumor immunotherapy.

## Abbreviations

PD-1: Programmed death receptor-1; PD-L1/2: Programmed death receptor-1 ligand1/2; TCR: T cell receptor; Lm: Listeria monocytogene; LLO: Listeriolysin-O; Treg: Regulatory T cell, MDSC- myeloid-derived suppressor cells; PAMP: Pathogen-associated molecular pattern; HPV16: Human papillomavirus strain 16; CFU: Colony-forming unit; DC: Dendritic cell; PBMC: Peripheral blood mononuclear cells; PE: Phycoerythrin; FITC: Fluorescein isothiocyanate; Ab: Antibody; CTL: Cytotoxic T lymphocyte; CPM: Cyclophosphamide.

## Competing interests

AW, RS and JR are current or former employees of Advaxis Inc. which provided Lm-LLO and Lm-LLO-E7. RS and JR are shareholders of Advaxis Inc. Other authors declare no conflict of interests.

## Authors’ contributions

MM and SK were the main investigators and take primary responsibility for the paper. MM performed the in vivo experiments and immune response analysis, RAE performed tumor infiltration analysis, NC carried out the in vitro experiments with dendritic cells. AW, RS and JR participated in the design and coordination, and helped to draft the manuscript. MM and SK wrote the paper. All authors read and approved the final manuscript.
